# GD2 or HER2 targeting T cell engaging bispecific antibodies to treat osteosarcoma

**DOI:** 10.1186/s13045-020-01012-y

**Published:** 2020-12-10

**Authors:** Jeong A. Park, Nai-Kong V. Cheung

**Affiliations:** Department of Pediatrics, Memorial Sloan Kettering Cancer Center, 1275 York Ave, Box 170, New York, NY USA

**Keywords:** Osteosarcoma, Immunotherapy, Bispecific antibody, Disialogangliosides, Human epidermal growth factor receptor-2, T cell arming, Ex vivo bispecific antibody-armed T cells (EATs), Programmed cell death protein 1 (PD-1), Programmed cell death-1 ligand 1(PD-L1)

## Abstract

**Background:**

The cure rate for metastatic osteosarcoma has not substantially improved over the past decades. Clinical trials of anti-HER2 trastuzumab or anti-GD2 dinutuximab for metastatic or refractory osteosarcoma were not successful, and neither was immune checkpoint inhibitors (ICIs).

**Methods:**

We tested various target antigen expressions on osteosarcoma cell lines using flow cytometry and analyzed in vitro T cell engaging BsAb (T-BsAb)-dependent T cell-mediated cytotoxicity using 4-h ^51^Cr release assay. We tested in vivo anti-tumor activities of T-BsAb targeting GD2 or HER2 in established osteosarcoma cell line or patient-derived xenograft (PDX) mouse models carried out in BALB-*Rag2*^−/−^IL-2R-*γc*-KO (BRG) mice. We also generated ex vivo BsAb-armed T cells (EATs) and studied their tumor-suppressive effect against osteosarcoma xenografts. In order to improve the anti-tumor response, ICIs, anti-human PD-1 (pembrolizumab) or anti-human PD-L1 (atezolizumab) antibodies were tested their synergy with GD2- or HER2-BsAb against osteosarcoma.

**Results:**

GD2 and HER2 were chosen from a panel of surface markers on osteosarcoma cell lines and PDXs. Anti-GD2 BsAb or anti-HER2 BsAb exerted potent anti-tumor effect against osteosarcoma tumors in vitro and in vivo. T cells armed with anti-GD2-BsAb (GD2-EATs) or anti-HER2-BsAb (HER2-EATs) showed significant anti-tumor activities as well. Anti-PD-L1 combination treatment enhanced BsAb-armed T cell function in vivo and improved tumor control and survival of the mice, when given sequentially and continuously.

**Conclusion:**

Anti-GD2 and anti-HER2 BsAbs were effective in controlling osteosarcoma. These data support the clinical investigation of GD2 and HER2 targeted T-BsAb treatment in combination with immune checkpoint inhibitors, particularly anti-PD-L1, in patients with osteosarcoma to improve their treatment outcome.

## Introduction

Osteosarcoma is the most common primary bone tumor in childhood and adolescence. With the introduction of multiagent chemotherapy, overall survival has improved to 60–70% [1]. However, survival rates have remained stagnant, and the prognosis for patients with metastatic or relapsed disease remains poor, with a 5-year overall survival rate of 20% [2–4]. Since the provocative observations of Dr. Coley on bacterial toxins inducing tumor regression [5], many immunotherapy attempts have been made in soft tissue and bone sarcomas, but success has been very limited [6, 7]. The EURAMOS-1 clinical trial, which incorporated IFN-α2b, failed to show the benefit [8], and also, antibody-based immunotherapies have not succeeded in improving outcome including trastuzumab or dinutuximab in clinical trials. Recent whole-genome sequencing (WGS) and molecular profiling studies of osteosarcoma have shown high levels of chromosome structural variations, rearrangements, and mutation clusters that result in significant disease heterogeneity but few clinically approachable alterations [9, 10]. These studies have yielded insights into aberrant signaling pathways such as PI3K/mTOR, IGF, and Wnt [11–13], but the efficacy of these targeted therapies in unselected high-risk osteosarcoma patients has been limited [14, 15].

On the other hand, exploiting cytotoxic T cells against osteosarcoma remains a viable alternative. Yet, upregulation of programmed cell death-1 receptor (PD-1) on CD8(+) tumor-infiltrating lymphocytes (TILs) and interaction with its ligands (PD-L1 and PD-L2) in tumor cells are proven immune escape routes to impede anti-tumor activity of T cells against osteosarcoma [16, 17]. Although immune checkpoint inhibitors (ICIs) have yet to demonstrate their benefit in patients with osteosarcoma (NCT02406781), blockade of PD-1 and PD-L1 interactions showed their potential to improve anti-tumor response in preclinical studies [18].

Here, we exploit bispecific antibody-directed T cell immunotherapy for osteosarcoma. We choose disialogangliosides (GD2) and human epidermal growth factor receptor-2 (HER2) as candidate target antigens because of their high expression across a number of osteosarcoma cell lines and their proven safety in IgG-mediated treatment of neuroblastomas and breast cancers using IgG monoclonal antibodies, respectively. We previously described T cell engaging bispecific antibodies (T-BsAbs) using sequences of anti-CD3 (huOKT3) and anti-disialoganglioside [GD2] (hu3F8) or anti-epidermal growth factor receptor-2 [HER2] (trastuzumab) antibody structured on IgG-[L]-scFv format with silenced Fc, exerting potent anti-tumor activities [19, 20]. Anticipating T cells in cancer patients to be suboptimal in cell number and function [21], arming ex vivo expanded T cells with T-BsAb should improve in vivo efficacy of BsAb treatment and minimize the risk of neurotoxicity or significant cytokine release syndrome (CRS), which was encountered by direct BsAb injection or CAR T cell treatment [22–25]. Here, we test anti-tumor activities of anti-GD2-BsAb and anti-HER2-BsAb against osteosarcoma in vitro and in vivo. In addition, we generate ex vivo armed T cells (EATs) using the anti-GD2-BsAb (GD2-EATs) or anti-HER2-BsAb (HER2-EATs) and evaluate their antitumor efficacy. Furthermore, we incorporate immune checkpoint inhibitors (ICIs), anti-human PD-1 (pembrolizumab), or anti-human PD-L1 (atezolizumab) antibodies to GD2-EAT or HER2-EAT therapy and study the optimal inhibitor and combination schedule in order to improve their anti-tumor response.

## Methods

### Cell lines

Representative human osteosarcoma cell lines, 143B (ATCC Cat# CRL-8304, RRID:CVCL_3477), U-2 OS (ATCC Cat# HTB-96, RRID:CVCL_0042), MG-63 (ATCC Cat# CRL-1427, RRID:CVCL_0426), HOS (ATCC Cat# CRL-1543, RRID:CVCL_0312), and Saos-2 (ATCC Cat# HTB-85, RRID:CVCL_0548), and osteoblast cell line, hFOB 1.19 (ATCC Cat# CRL-11372, RRID:CVCL_3708), were purchased from ATCC (Manassa VA). All cells were authenticated by short tandem repeats profiling using PowerPlex 1.2 System (Promega, Cat# DC8942), and periodically tested for mycoplasma infection using a commercial kit (Lonza, Cat# LT07-318). The cells were cultured in RPMI1640 (Sigma) supplemented with 10% heat-inactivated fetal bovine serum (FBS, Life Technologies) at 37 °C in a 5% CO_2_ humidified incubator.

### Flow cytometry

For flow cytometric analysis of antigen expression by human osteosarcoma cell lines, cells were harvested, and cell viability was determined. 1 × 10^6^ cells were stained with 1 μg of antigen specific mAbs in a total volume of 100 μL for 30 min at 4 °C. Anti-CD20 chimeric mAb, rituximab, or mouse IgG1 monoclonal antibody was used as isotype control. After washing with PBS, cells were re-incubated with 0.1 µg PE-conjugated goat anti-human IgG Ab (SouthernBiotech Cat# 2040-09, RRID:AB_2795648). For each sample, 20,000 live cells were analyzed using a BD FACS Calibur TM (BD Biosciences, Heidelberg, Germany). Data were analyzed with FlowJo V10 software (FlowJo, RRID:SCR_008520) using geometric mean fluorescence intensity (MFI). The MFI for isotype control antibody was set to 5, and the MFIs for antibody binding were normalized based on isotype control.

### Effector cell preparation

Effector peripheral blood mononuclear cells (PBMC) were separated by Ficoll from buffy coats purchased from the New York Blood Center. T cells were purified from PBMC using Pan T cell isolation kit (Miltenyi Biotec, Cat# 130096535). These T cells were activated by CD3/CD28 Dynabeads (Gibco™, Cat# 11132D) for 7 to 14 days in the presence of 30 IU/mL of IL-2 according to manufacturer’s protocol. PBMCs and ATCs were analyzed by FACS for their proportion of CD3(+), CD4(+), CD8(+), and CD56(+) cells.

### Cytotoxicity assays (^51^chromium release assay)

Antibody-dependent T cell-mediated cytotoxicity (ADTC) was assessed by ^51^Cr release assay, and EC_50_ was calculated using Sigma Plot software. Tumor cells were labeled with sodium ^51^Cr chromate (Amersham, Arlington Height, IL) at 100 mCi/10^6^ cells at 37^◦^C for 1 h. After two washes, tumor cells were plated in a 96-well plate before mixing with activated T cells (ATCs) at decreasing concentrations of T-BsAb. Effector-to-target cell ratio (ET ratio) was 10:1, and cytotoxicity was analyzed after incubation at 37^◦^C for 4 h. The released ^51^Cr was measured by a gamma counter (Packed Instrument, Downers Grove, IL). Percentage of specific lysis was calculated using the formula: 100% (experimental cpm—background cpm)/(total cpm—background cpm), where cpm represented counts per minute of ^51^Cr released. Total release of ^51^Cr was assessed by lysis with 10% SDS (Sigma, St Louis, Mo, Cat# 71736), and background release was measured in the absence of effector cells and antibodies.

### Antibodies

For each BsAb, scFv of huOKT3 was fused to the C-terminus of the light chain of human IgG1 via a C-terminal (G4S)3 linker [26]. N297A and K322A on Fc were generated with site-directed mutagenesis via primer extension in polymerase chain reactions [27]. The nucleotide sequence encoding each BsAb was synthesized by GenScript and was subcloned into a mammalian expression vector. Each BsAb was produced using Expi293™ expression system (Thermo Fisher Scientific, Cat# A14635) separately. BsAbs were purified with protein A affinity column chromatography. GD2-BsAb was linked to the carboxyl end of the anti-GD2 hu3F8 IgG1 light chain [19], and HER2-BsAb linked to the anti-HER2 trastuzumab IgG1 light chain [20]. Anti-CD33/anti-CD3 BsAb or anti-GPA33/anti-CD3 BsAb was used as a control BsAb for ADTC assay and in vivo animal experiments [28, 29]. The other BsAbs used in this study were previously described (US patent# 62/896,415). The purity of BsAbs was evaluated by size-exclusion high-performance liquid chromatography (SE-HPLC), and they had high levels of purity (> 90%). The BsAbs remained stable by SDS-PAGE and SEC-HPLC after multiple freeze–thaw cycles. The biochemical data of BsAbs used are summarized in Additional file 1: Table S1.

### T cell arming

Ex vivo activated T cells were harvested between day 7 and day 14 and armed with each BsAb for 20 min at room temperature. After incubation, the T cells were washed with PBS twice. Properties of ex vivo bispecific antibody-armed T cells (EATs) were tested for cell surface density of BsAb using flow cytometry and in vitro cytotoxicity against target antigens. BsAb bound to T cell was measured with anti-idiotype antibody for GD2-EATs and anti-human IgG Fc antibody (BioLegend, Cat# 409303, RRID:AB_10900424) for HER2-EATs.

### In vivo experiments

All animal experiments were approved by the Memorial Sloan Kettering’s Institutional Animal Care and Use Committee (IACUC) and were executed according to the IACUC guidelines. For in vivo experiments, BALB-*Rag2*^−^/^−^IL-2R-*γc*-KO (BRG) mice (Taconic Biosciences) were used [30]. In vivo experiments were performed in 6–10-week-old male mice. Tumor cells were suspended in Matrigel (Corning Corp, Tewksbury MA) and implanted in the flank of BRG mice. Besides tumor cell line xenografts, 3 different patient-derived tumor xenografts (PDXs) positive for both GD2 and HER2 were established from fresh surgical specimens with MSKCC IRB approval. Tumor size was measured using TM900 scanner (Piera, Brussels, BE), and treatment was started when tumor size reached 100 mm^3^. Before treatment, mice were randomly assigned to each group. Tumor growth curves and overall survival were analyzed, and the overall survival was defined as the time from the start of treatment to when tumor volume reached 2000 mm^3^. To define well-being of mice, CBC analyses, changes in body weight, general activity, physical appearance, and GVHD scoring were monitored. All animal experiments were repeated twice more with different donor’s T cells to ensure that our results were reliable.

### Cytokine release assays

Human Th1 cell-released cytokines were analyzed after EAT injection using LEGENDplexTM Human Th1 Panel (Biolegend, Cat# 741035). Five human T cell cytokines including IL-2, IL-6, IL-10, IFN-γ, and TNF-α were analyzed using mouse serum 4 h, 12 h, and 24 h after EAT injection.

### Single cell suspension for flow cytometry analysis of tumor

Surgically resected tumor samples were transported in PBS at room temperature and transferred to 50-mL conical tubes with warm medium (RPMI1640 + 10% FBS). Tissues were dissociated to 1–3 mm^3^ pieces using scalpels with blade and followed by 1-h enzymatic dissociation using 20X Collagenase II (ThermoFisher Scientific, Cat# 17101015), 100X DNase I (ThermoFisher Scientific, Cat# EN0521). Samples were filtered with 70-µm and 40-µm cell strainers, and red blood cells were eliminated using ACK lysis buffer (ThermoFisher Scientific, Cat# A1049201). After centrifugation, cells were resuspended in warm medium and counted to quantify viable cells using Trypan blue.

### Flow cytometry of blood and tumor

Peripheral blood and tumors were collected and analyzed by flow cytometry. Anti-human antibodies against CD3 (BioLegend, Cat# 300308, RRID:AB_314044), CD4 (BioLegend, Cat# 357410, RRID:AB_2565662), CD8 (BioLegend, Cat# 300912, RRID:AB_314116), and CD45 (BioLegend, Cat# 304012, RRID:AB_314400) were used to define T cell engraftment and subpopulation, and anti-human PD-1 (BioLegend Cat# 367410, RRID:AB_2566680) and PD-L1 antibodies (BioLegend Cat# 329706, RRID:AB_940368) were used to quantify their expression by T cells and osteosarcoma tumor cells. Stained cells were processed with BD LSRFORTESSA (BD Biosciences, Heidelberg, Germany) and analyzed with FlowJo software (FlowJo, LLC, Ashland, OR).

### Immunohistochemical (IHC) staining

Formalin-fixed paraffin-embedded tumor sections were made, and immunohistochemical (IHC) staining for human CD3, CD4, and CD8 T cells was done to confirm T cell infiltration inside tumors. The IHC staining was performed at Molecular Cytology Core Facility of MSKCC using Discovery XT processor (Ventana Medical Systems). Paraffin-embedded tumor sections were deparaffinized with EZPrep buffer (Ventana Medical Systems), antigen retrieval was performed with CC1 buffer (Ventana Medical Systems), and sections were blocked for 30 min with background buffer solution (Innovex). Anti-CD3 (Agilent, Cat# A0452, RRID:AB_2335677, 1.2 μg/mL), anti-CD4 (Ventana Medical Systems Cat# 790-4423, RRID:AB_2335982, 0.5 μg/mL), and anti-CD8 (Ventana Medical Systems Cat# 790-4460, RRID:AB_2335985, 0.07 μg/mL) were applied, and sections were incubated for 5 h, followed by 60-min incubation with biotinylated goat anti-rabbit IgG (Vector Laboratories, Cat# BA-1000, RRID:AB_2313606) at 1:200 dilution. For PD-L1 staining, the sections were pre-treated with Leica Bond ER2 Buffer (Leica Biosystems) for 20 min at 100 °C, stained with PD-L1 rabbit monoclonal antibody (cell signaling, Cat# 29122, 2.5 mg/mL) for 1 h on Leica Bond RX (Leica Biosystems). Control antibody staining was done with biotinylated goat anti-rat IgG (Vector Laboratories, Cat# BA-9400, RRID:AB_2336202). All images were captured from tumor sections using Nikon ECLIPSE Ni-U microscope and NIS-Elements 4.0 imaging software. Antigen-positive cells were counted with Qupath 0.1.2.

### GD2 expression by IHC

Fresh-frozen tumor sections were made using Tissue-Tek OCT (Miles Laboratories, Inc, Elkhart, IN) with liquid nitrogen and stored at − 80 °C. The tumor sections were stained with mouse IgG3 mAb 3F8 as previously described [31]. Stained slides were captured using a Nikon ECLIPSE Ni-U microscope and analyzed, and the tissue staining intensity was compared with positive and negative controls and scored from 0 to 4 according to two components: staining intensity and percentage of positive cells. Each sample was assessed and graded by two independent observers.

### Statistics

In vivo anti-tumor effect and cytokine release analyses were compared using area under curve (AUC) and survival curves calculated using GraphPad Prism 8.0. Differences between samples indicated in the figures were tested for statistical significance by two-tailed Student’s t-test for two sets of data, while one-way ANOVA was used to among three or more sets of data. All statistical analyses were performed using GraphPad Prism V.8.0 for Windows (GraphPad Prism, RRID:SCR_002798). *P* < 0.05 was considered statistically significant. Asterisks indicate that the experimental *P-*value is statistically significantly different from the associated controls at * *P* < 0.05; ** *P* < 0.01; *** *P* < 0.001, **** *P* < 0.0001.

## Results

### GD2 and/or HER2 was overexpressed on majority of osteosarcoma cell lines

To identify potential therapeutic targets for osteosarcoma, we conducted a literature review and assessed the expression of surface target antigens, GD2, GD3, HER2, B7H3 (CD276), high molecular weight melanoma antigen (HMW-M), gene name chondroitin-sulfate proteoglycan-4 (CSPG4), glycoprotein A33 (GPA33), L1 cell adhesion molecule (L1CAM), glypican-3 (GPC-3), Lewis Y, prostate-specific antigen (PSA), Globo H, interleukin 11 receptor-α (IL-11Rα), PD-L1, prostate-specific membrane antigen (PSMA), and insulin-like growth factor 2 receptor (IGF2R), reported to be overexpressed by osteosarcoma [32–35]. Surface antigen expression levels were semiquantitated by flow cytometric analysis and normalized with the geometric mean fluorescence intensity (MFI) of a control antibody (Table 1 and Additional file 2: Fig. S1). The majority of osteosarcoma cell lines overexpressed GD2 and/or HER2 antigen on their cell surface. However, their MFIs for GD2 or HER2 staining were generally much lower than those for GD2(+) neuroblastoma cell lines, or HER2(+) breast cancer cell lines, respectively. Based on their MFIs, GD2, HER2, B7H3, CSPG4, L1CAM (CD171), and Lewis Y were chosen as tumor targets for further in vitro screening.

**Table 1:** Tumor-associated antigen expression (MFI, mean fluorescence intensity) in osteosarcoma

Cell line	Isotype control	GD2	GD3	HER2	B7H3 (CD276)	HMW-M CSPG4	GPA33	L1CAM	GPC-3	Lewis Y	PSA	Globo H	IL-11Ra	PD-L1	PSMA	IGF2R
Osteosarcoma																
143B	5	64	9	32	261	83	6	24	6	16	8	7	14	36	6	7
U-2 OS	5	135	10	75	255	124	5	49	15	41	7	8	18	44	5	8
MG-63	5	45	10	30	346	66	6	24	8	18	14	6	7	89	5	8
HOS	5	11	11	38	268	40	8	21	14	27	10	7	18	69	5	8
Saos-2	5	9	6	54	357	37	8	23	14	43	10	6	22	124	5	10
hFOB1.19	5	10	6	76	724	55	5	40	8	38	6	6	17		5	
Neuroblastoma																
IMR32	5	1468														
LAN-1	5	1625														
BE(2)C	5	1005														
Breast cancer																
HCC1594	5			2091												
SKBR3	5			2506												
AU565	5			1175												

GD2, disialoganglioside GD2; GD3, disialohematoside; HER2, human epidermal growth factor receptor 2; HMW, high molecular weight melanoma antigen; CSPG4, chondroitin-sulfate proteoglycan 4; GPA, glycoprotein A33; L1CAM, L1 cell adhesion molecule; GPC-3, glypican-3; PSA, polysialic acid; PD-L1, programmed death-ligand 1; PSMA, prostate-specific membrane antigen; IGF2R; insulin-like growth factor 2 receptor

### GD2-BsAb and HER2-BsAb exerted strong cytotoxicity against osteosarcoma cell lines in vitro

**Fig. 1:**
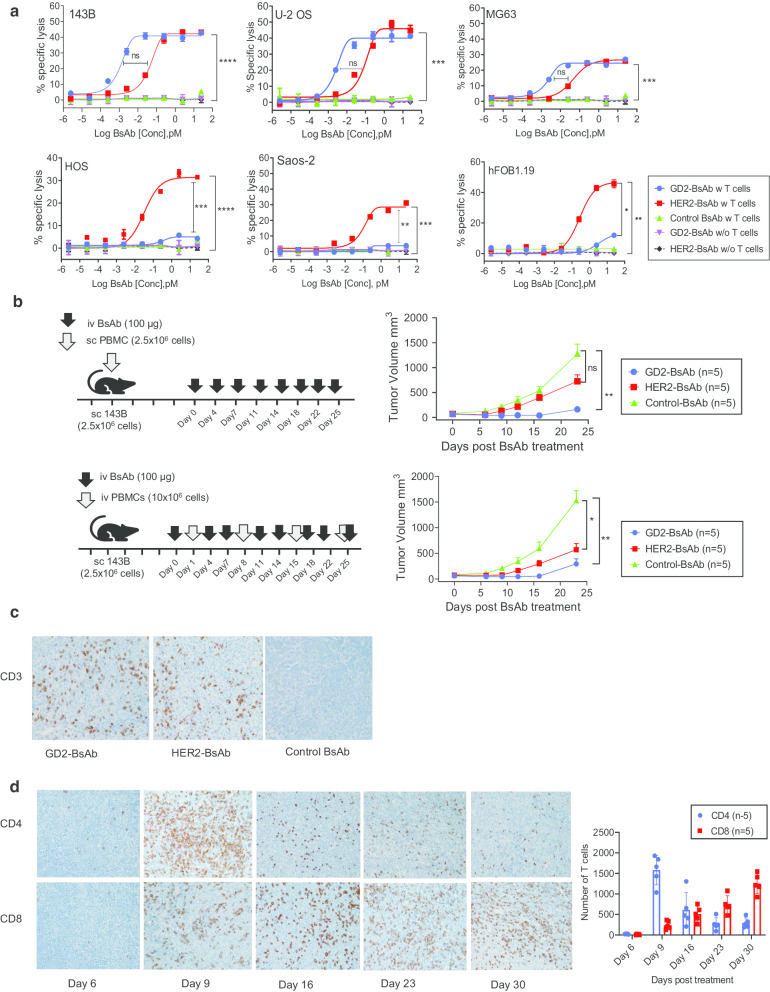
GD2-BsAb and HER2-BsAb exerted strong cytotoxicity against osteosarcoma. **a** Antibody-dependent T cell-mediated cytotoxicity (ADTC) was analyzed by ^51^Cr release assay using activated T cells (ET ratio, 10 to 1) at decreasing concentrations of BsAb in an osteosarcoma cell line panel. Cytotoxicity was compared to control BsAb with T cells and each BsAb alone. **b** In vivo anti-tumor activity of GD2-BsAb and HER2-BsAb against osteosarcoma*.* **c** Immunohistochemical (IHC) staining of tumor-infiltrating lymphocytes. Tumors were harvested on day 30 post-treatment and stained with anti-human CD3 antibody (× 200). **d** IHC staining of tumors by anti-human CD4 and anti-human CD8 antibody (× 200) following treatment with iv PBMC and iv GD2-BsAb

Antibody-dependent T cell-mediated cytotoxicity (ADTC) assay using activated T cells (E:T ratio of 10:1) in the presence of decreasing concentrations of BsAbs [1 μg/mL (5 nM) and serial tenfold dilutions] was performed against a panel of osteosarcoma cell line. All tested BsAbs were made using the IgG-[L]-scFv format with silenced Fc, and anti-CD33/anti-CD3 BsAb was used for control Ab [29]. Among them, anti-GD2 BsAb (GD2-BsAb)and anti-HER2-BsAb (HER2-BsAb) showed the most potent ADTC against the panel of osteosarcoma cell lines (Table 2). For GD2-BsAb, cytotoxicity was robust (EC50 of 0.2 to 0.5 pM) for GD2(+) osteosarcoma cell lines (143B, U-2 OS, and MG-63), where maximal killing was observed between 5 and 500 pM (Fig. 1a). HER2-BsAb also mediated potent ADTC against most of the osteosarcoma cell lines which were HER2-positive (143B, U-2 OS, MG-63, HOS, and Saos-2) and against hFOB1.19, with maximal cytotoxicity at 5 to 500 pM. Anti-tumor potency of each BsAb [EC50 (a measure of in vitro sensitivity to ADTC)] was inversely correlated with MFIs of each target antigen. Although B7H3, L1CAM, CSPG4, and Lewis Y were also overexpressed by some osteosarcoma cell lines, the ADTC potency of their respective BsAb was much weaker than GD2-BsAb or HER2-BsAb. Based on these findings, the targets GD2 and HER2 were chosen for further in-depth T cell-based immunotherapy studies.

**Table 2:** In vitro sensitivities (EC50, pM) to target antigen-specific bispecific antibodies in osteosarcoma cell lines

	GD2	HER2	B7H3	CSPG4	L1CAM	Lewis Y
Osteosarcoma						
143B	0.2	10	130	454	329	1440
U-2 OS	0.5	11	150	116	92	692
MG-63	0.4	8	109	558	397	4655
HOS	3906	10	274	645	158	312
Saos-2	> 5000	18	120	500	229	468
Osteoblast						
hFOB 1.19	> 5000	69	126	498	121	600

### GD2-BsAb and HER2-BsAb showed potent anti-tumor activities against osteosarcoma xenografts

Next, we tested in vivo anti-tumor response of GD2-BsAb and HER2-BsAb against osteosarcoma xenografts (Fig. 1b). In the first xenografted mouse model, osteosarcoma 143B tumor cells were mixed with PBMCs and implanted subcutaneously (sc) into mice. Tumors were treated with intravenous (iv) GD2-BsAb or HER2-BsAb twice per week for 4 weeks. GD2-BsAb and HER2-BsAb suppressed osteosarcoma growth compared to negative controls (GPA33-BsAb) (*P* = 0.0005 for GD2-BsAb and *P* = 0.10 for HER2-BsAb, respectively). This finding was reproduced in a second tumor model where PBMCs were administered weekly iv instead of sc. Both GD2-BsAb and HER2-BsAb significantly suppressed tumor growth compared to controls (*P* = 0.0025 for GD2-BsAb and *P* = 0.0248 for HER2-BsAb, respectively).

To confirm this anti-tumor effect, T cell infiltration inside tumors was studied using IHC staining after treatment of GD2-BsAb and HER2-BsAb with iv PBMCs. CD3(+) TILs were detected in both GD2-BsAb- and HER2-BsAb-treated tumors (Fig. 1c), but not in tumors treated with control BsAb. Serial T cell infiltration was also investigated by staining tumors on days 6, 9, 16, 23, and 30 post-treatment (Fig. 1d). While TILs showed CD4(+) T cell predominance on day 9, CD8 (+) T cells became predominant at later time points (day 23 and day 30).

### Adoptive T cell immunotherapy using ex vivo armed T cells (EATs) carrying GD2-BsAb or HER2-BsAb was effective

To optimize BsAb treatment, ex vivo GD2-BsAb-armed T cells (GD2-EATs) and HER2-BsAb-armed T cells (HER2-EATs) were generated, and we assessed their safety and efficacy for the treatment of osteosarcoma. Both EATs showed stable BsAb binding (Additional file 2: Fig. S2A) and effective tumor cell killing against osteosarcoma cell lines over a range of E:T ratios and antibody doses (Additional file 2: Fig. S2B). Maximum killing was observed between 0.05 μg and 5 μg /10^6^ T cells of BsAb-arming concentration. To test the stability of EATs, GD2-EATs and HER2-EATs were incubated at 4℃ for 72 h, and the MFIs of each BsAb were measured using anti-human IgG Fc antibody at different time points. Both GD2-EATs and HER2-EATs showed stable BsAb binding over time, and their MFIs after 72 h have maintained 80% of the levels of EATs at first (Additional file 2: Fig. S2C).

**Fig. 2:**
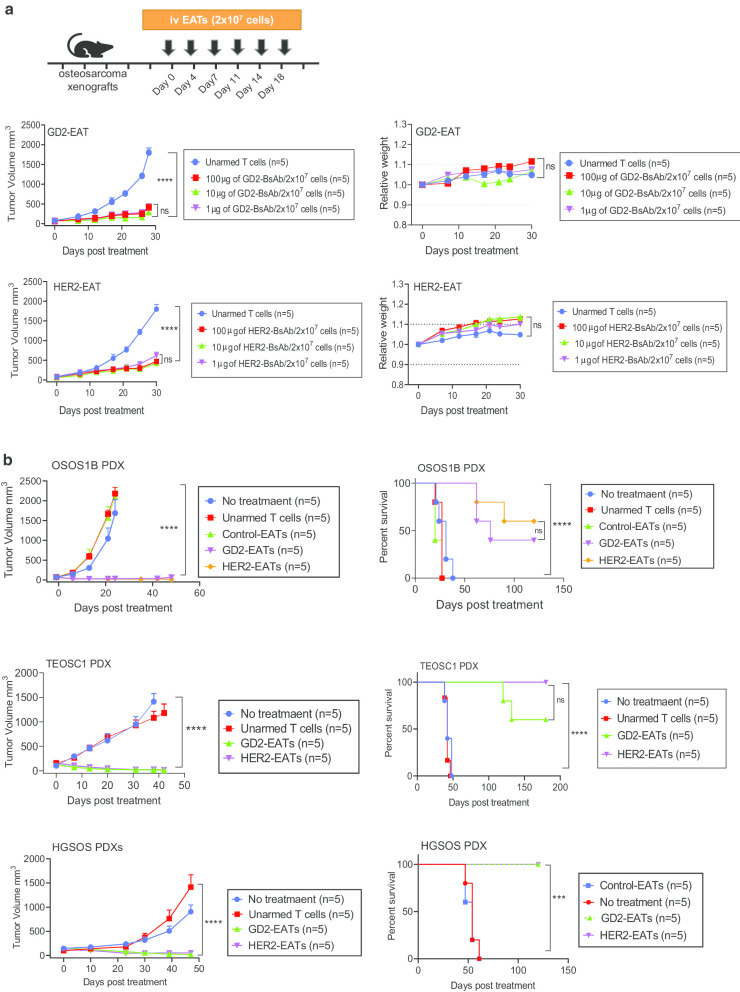
In vivo anti-tumor activity of ex vivo GD2-BsAb or HER2- BsAb-armed T cells (EATs). **a** Anti-tumor activities of GD2-EATs and HER2-EATs were tested over a range of BsAb arming concentrations in vivo. **b** GD2-EATs (10 μg of GD2-BsAb/2 × 10^7^ cells) and HER2-EATs (10 μg of HER2-BsAb/2 × 10^7^ cells) exerted a potent anti-tumor effect against a variety of osteosarcoma PDXs without significant toxicity, improving survival

To address in vivo anti-tumor effect and safety of EATs*,* we treated 143B xenografts with 2 × 10^7^ of EATs armed with increasing concentrations (1 to 100 µg) of GD2-BsAb or HER2-BsAb (Fig. 2a). In vivo cytokine levels were analyzed following EATs or unarmed T cells injection (Additional file 2: Fig.S3). Although high-dose GD2-EATs (100 μg/2 × 10^7^ cells) released higher levels of IL-2 and TNF-α compared to controls, TH1 cell cytokines (except IFN-γ) were not significantly elevated after EATs injection. Only IFN-γ levels were significantly elevated in GD2-EAT-treated mice compared to controls. Most mice maintained their body weight and activity and did not exhibit toxicity during the follow-up period. Tumor growth was significantly suppressed over a range of BsAb-arming concentrations (1 to 100 µg of BsAb/2 × 10^7^ cells) in contrast to the control group (2 × 10^7^ of unarmed T cells) (*P* < 0.0001). This potent anti-tumor effect of GD2-EATs and HER2-EATs was confirmed in multiple osteosarcoma PDX mouse models (Fig. 2b). GD2-EATs and HER2-EATs successfully regressed multiple osteosarcoma PDX tumors, leading to significantly improved survival compared to negative controls [no treatment, unarmed T cells, or control EATs (T cells armed with GPA33-BsAb)], (*P* < 0.0001).

### Combinational treatment of immune checkpoint inhibitors and GD2- or HER2-bispecific antibody-armed T cell immunotherapy

**Fig. 3:**
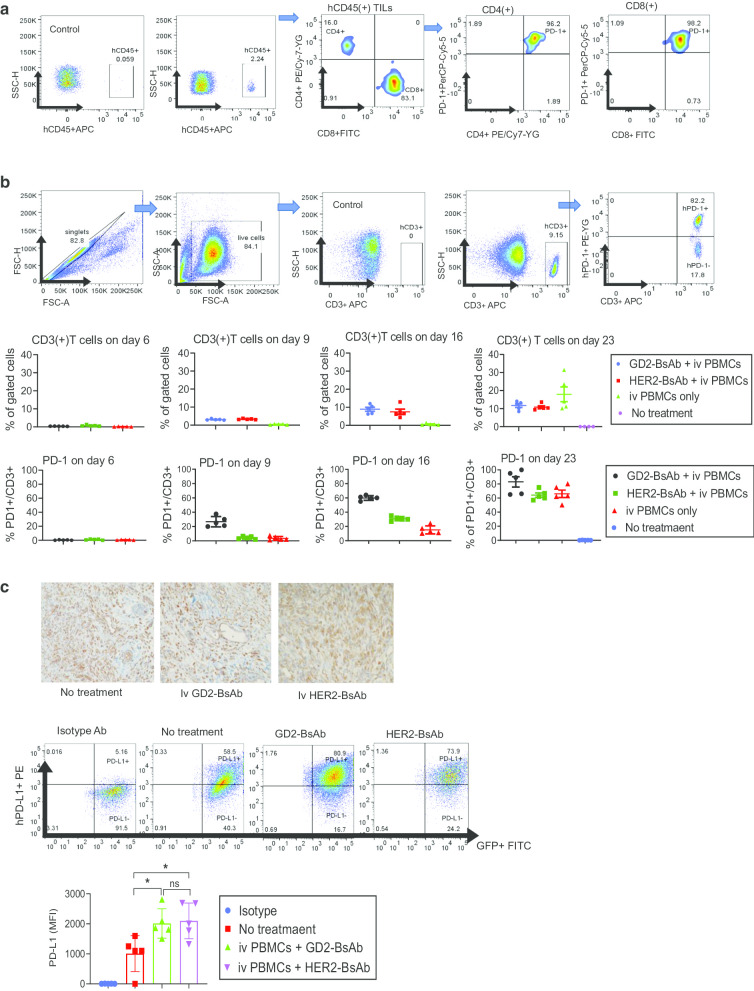
PD-1 and PD-L1 expression by T cells and osteosarcoma cell line xenografts. **a** Flow cytometry analysis of PD-1 expression on tumor-infiltrating lymphocytes (TILs) in osteosarcoma 143B cell line xenografts on day 35 post-GD2-BsAb treatment. **b** Flow cytometry analyses of human CD3(+) T cells and human PD-1 expression by CD3(+) T cells in peripheral blood after GD2-BsAb or HER2-BsAb treatment. **c** IHC staining and flow cytometry analysis of human PD-L1 in osteosarcoma 143B xenografts. PD-L1 expression levels were quantified using geometric MFI (MFI)

Although GD2-BsAb and HER2-BsAb recruited substantial numbers of T cells into tumors and successfully suppressed tumor growth compared to controls, some tumors showed resistance or relapsed following the initial response. TILs showed predominance of CD8(+) T cells, and the majority of which expressed PD-1 on their surface (Fig. 3a). Circulating CD3(+) T cells in peripheral blood on days 6, 9, 16, and 23 post-treatment showed gradual increase of PD-1 expression from less than 5% to over 75% after treatment with GD2-BsAb, being different from controls (iv PBMCs only) which showed a more sudden surge of circulating T cells and PD-1 expression on day 23 post-treatment, which attributed to human hematopoietic and lymphoid cell engraftment and xenogeneic graft-versus-host disease (Fig. 3b) [36, 37]. In addition to PD-1 expression on T cells, osteosarcoma xenografts were PD-L1-positive by IHC staining and FACS analyses, and PD-L1 expression was upregulated following BsAb treatment (Fig. 3c).

### PD-L1 blockade augmented anti-tumor activity of EAT therapy

**Fig. 4:**
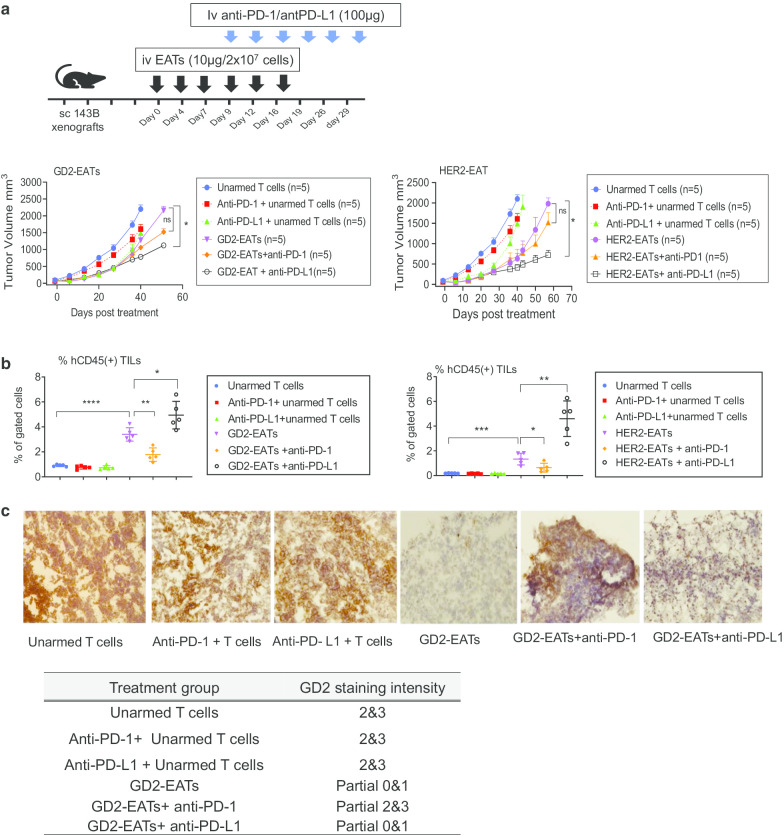
Combination of immune checkpoint inhibitors with BsAb treatment. **a** In vivo anti-tumor effect of PD-1 blockades and GD2-EATs or HER2-EATs combination therapy against osteosarcoma. **b** Flow cytometric analyses of tumor-infiltrating lymphocytes (TILs) on day 52 (for GD2-EATs) and day 58 (for HER2-EATs) post-treatment. **c** Fresh-frozen tumor sections were stained with mouse IgG3-3F8, and GD2 expression was scored by staining intensity

To test whether PD-1 blockades can overcome T cell exhaustion related to treatment resistance, we combined anti-PD-1 (pembrolizumab) or anti-PD-L1 (atezolizumab) monoclonal antibody and GD2-EATs or HER2-EATs and evaluated their synergy in osteosarcoma cell line xenograft mouse model (Fig. 4a). GD2-EATs or HER2-EATs were administered twice a week for 3 weeks, and iv anti-PD-1 or anti-PD-L1 was started on day 9 post-EAT therapy and given twice per week for 3 weeks, based on the anticipated PD-1 upregulation in T cells by day 9 (Fig. 3b). Anti-PD-L1 plus GD2-EATs or HER2-EATs improved tumor control compared to EATs alone (*P* = 0.0257 for GD2-EATs and *P* = 0.0374 for HER2-EATs), while combination with anti-PD-1 failed to improve the anti-tumor response compared to GD2-EATs or HER2-EATs alone (*P* = 0.1969 and *P* = 0.7894, respectively). Anti-PD-L1 combination resulted in greater frequencies of T cells in tumors compared to GD2-EATs or HER2-EATs alone, whereas anti-PD-1 combination did not (Fig. 4b). Interestingly, GD2-EATs and GD2-EATs plus anti-PD-L1 appeared to eliminate GD2^high^ tumor cells while leaving GD2^low^ tumor cells behind (by IHC), but GD2-EATs plus anti-PD-1 did not show such effects (Fig. 4c), consistent with the lack of benefit from anti-PD-1 combination with EAT therapy.

### Timing of anti-PD-L1 during GD2-EAT therapy affected anti-tumor immune response

**Fig. 5:**
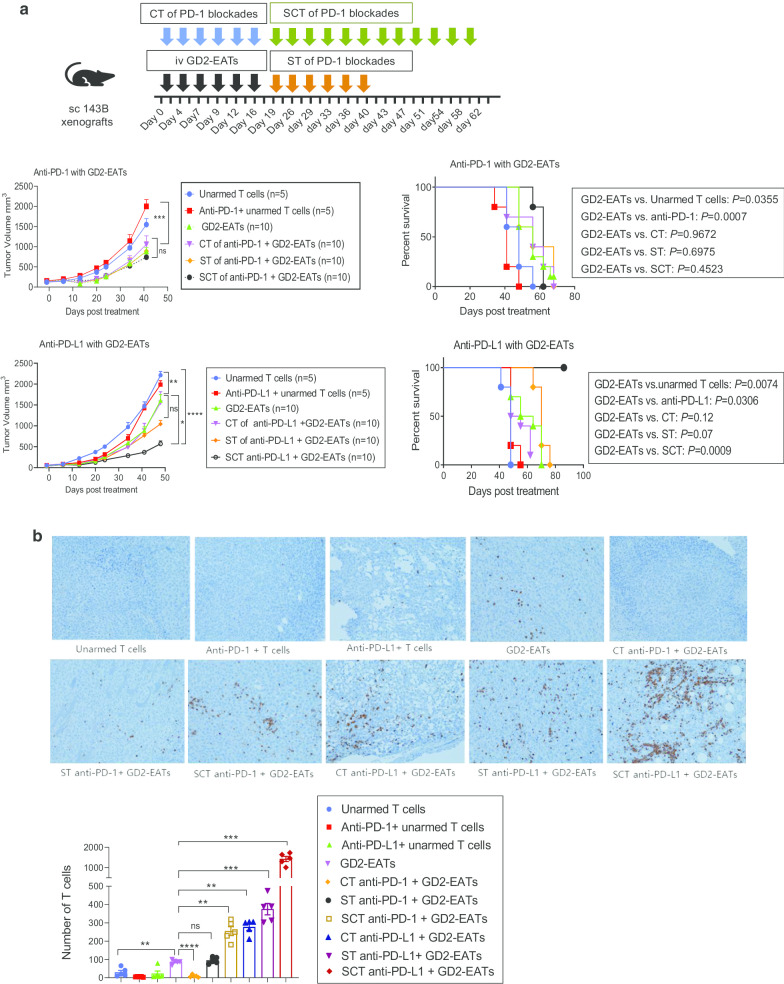
Optimal timing of anti-PD-L1 for combination treatment with GD2-BsAb. **a** Combination treatment of GD2-EATs and PD-1 blockades: a comparison of 3 different combination schedules of PD-1/PD-L1 antibody [concurrent therapy (CT) vs. sequential therapy (ST) vs. sequentially continuous therapy (SCT)]. **b** Formalin-fixed paraffin-embedded (FFPE) tumor sections of each group were stained with anti-human CD3 antibody (×200). CD3(+) T cell numbers were compared

Given the upregulation of PD-1/PD-L1 pathway following BsAb treatment, we tested three different time schedules of PD-1 blockades with GD2-EAT therapy (Fig. 5a). GD2-EATs were given three times per week for 2 weeks. Six doses of anti-PD-1 or anti-PD-L1 were given either (1) concurrently (concurrent therapy, CT), (2) sequentially after 6 doses of EATs (sequential therapy, ST), or (3) sequentially continuous way [additional 6 more doses of PD-1 blockades post-ST (sequentially continuous therapy, SCT)]. Combination with anti-PD-1 had no benefit, either using CT, ST or SCT regimens when compared to GD2-EATs alone. CT of anti-PD-L1 also failed to improve the anti-tumor response of GD2-EATs. However, anti-PD-L1 given as ST slowed the tumor growth, and SCT of anti-PD-L1 significantly suppressed tumor growths compared to GD2-EATs alone (*P* = 0.0149). While none of the anti-PD-1 combination regimens did improve survival over GD2-EATs, SCT of anti-PD-L1 did improve the survival of the mice compared to GD2-EATs alone (*P* = 0.0009).

To address the effect of PD-1 blockades on T cell infiltration into tumors, we harvested tumors when they reached 2000 mm^3^ or on the last day of the experiment. TILs were analyzed by flow cytometry (Additional file 2: Fig.S4A). GD2-EATs recruited more T cells into the tumors compared to unarmed T cells (*P* = 0.0295) or anti-PD-1 plus unarmed T cells (*P* = 0.0236). CT of anti-PD-1 resulted in fewer TILs than GD2-EATs (*P* = 0.0194). With ST regimen, anti-PD-1 showed comparable TIL frequency with GD2-EATs (*P* = 0.54); with SCT regimen, anti-PD-1 increased TIL frequency over GD2-EATs alone (*P* = 0.0056) (Additional file 2: Fig. S4B). On the other hand, CT of anti-PD-L1 did not affect TIL frequencies of GD2-EATs (*P* = 0.8815), but ST and SCT of anti-PD-L1 increased TIL frequencies over GD2-EATs alone (*P* = 0.0018 and *P* = 0.0005) (Additional file 2 Fig.S3C). Among the TIL subsets, tumors treated with SCT regimen (irrespective of anti-PD-1 or anti-PD-L1) had significantly greater frequencies of CD8(+) T cells compared to GD2-EATs alone (*P* < 0.0001). The difference in TIL frequencies among groups was confirmed by IHC staining using anti-CD3 antibody (Fig. 5b). Anti-PD-L1 combinations significantly increased CD3(+) T cell numbers in tumors compared to GD2-EATs alone (*P* < 0.0001) and consistently had greater frequencies of TILs than anti-PD-1 combinations, providing a rationale for combining with anti-PD-L1 rather than anti-PD-1 for synergy with BsAb-based T cell immunotherapy.

## Discussion

Osteosarcoma tissues overexpress GD2 and HER2 on their surface, and these antigens targeting strategies have been a subject of great attention. However, clinical trials of anti-HER2 trastuzumab or anti-GD2 dinutuximab for metastatic or refractory osteosarcoma were not successful [38, 39]. This failure attributed to relatively low expression levels of GD2 or HER2 on osteosarcoma tumor tissues [40], or inherent insensitivity of this tumor to Fc-dependent immune cytotoxic mechanisms [41, 42]. In this study, we targeted these antigens using T cell engaging BsAb and studied the anti-tumor effect of GD2-BsAb and HER2-BsAb against osteosarcoma. Both BsAbs successfully directed T cells into tumor tissues and exerted a significant anti-tumor effect. T cells armed with GD2-BsAb or HER2-BsAb showed potent tumor-suppressive effect in a variety of osteosarcoma xenograft mouse models with minimal in vivo toxicities. Moreover, osteosarcoma PDX-bearing mice showed long-term cures after GD2-EATs and HER2-EATs treatment, consistent with their high potency, although xenogeneic GVHD effect or epitope spread among long-term memory T cells in vivo cannot be ruled out [37, 43, 44]. The use of bispecific murine antibodies in syngeneic mouse models will help address these potential mechanisms of tumor control.

To improve therapeutic efficacy of GD2-EATs and HER2-EATs, combination with PD-1 blockades was tested. CD8(+) TILs in metastatic osteosarcoma tissues overexpressed PD-1, and PD-1/PD-L1 blockades partially improved T cell function, resulting in longer survival with fewer pulmonary metastases in previous studies [18]. However, how to optimally combine ICIs with other immunotherapies has yet to be determined, given the potential negative impact of the concurrent use of immunotherapeutics [45, 46]. Our data also showed that concurrently administered anti-PD-1 or anti-PD-L1 had no benefit. Sequentially continuous anti-PD-L1 only did improve the anti-tumor effect of GD2-EATs against osteosarcoma. It suggests that continuous neutralization of PD-L1 may be necessary for optimal synergy with BsAb and T cell immunotherapy.

Although cytotoxic CD8(+) T cells mediate direct tumor cell killing, CD4(+) T helper (TH) cells are also important in tumor cell eradication [47], as CD4(+) CAR T cells exert significant cytotoxicity comparable to CD8(+) CAR T cells [48]. According to recently published single-cell analysis data, both CD4(+) TH cells and CD8(+) cytotoxic T cells are equally effective in direct tumor cell killing, and their cytotoxicity is associated with both TH1 and TH2 cytokines, e.g., IFN-γ, TNF-α, IL-15, and IL-13, as confirmed by the expression of master transcription factor genes TBX21 and GATA3 [49, 50]. In addition, rather than stringent TH1 or TH2 subtypes, the predominant anti-tumor response is dependent on a highly mixed TH1/TH2 function in the same cell, suggesting the activation of BsAb-directed T cells or CAR T cells is a canonical process that leads to a mixed response combining both TH1 and TH2 cytokines together with GM-CSF [49]. This is consistent with the finding that polyfunctional CAR T cells are highly correlated to objective response of patients [51]. On the other hand, the ratio of CD4(+) to CD8(+) T cells does have an effect on the anti-tumor activity of CAR T cells [52, 53]. Furthermore, balanced ratio of CD4(+) and CD8(+) CAR T cells (CD4:CD8 ratio 1:1) seemed to be important for high remission rates in B-ALL [51]. CD4(+) T cells help CD8(+) T cells differentiate, recruit and expand through IL-2, IL-21 and other cytokines to perform their tumoricidal functions [54, 55]. CD4(+) T cells in tumorous condition or chronic infection are skewed toward the T follicular helper (TFH) phenotype [56, 57]. While the majority of exhausted T cells in tumors express intermediate levels of PD-1, TFH cells express high levels of PD-1, not predictive of patient survival [58, 59]. The in vivo anti-tumor effect was likely dependent on both CD4(+) T cells and CD8(+) T cells, as our serial IHC data (Fig. 1d) did suggest a temporal sequence where the initial arrival of CD4(+) T cells was followed by subsequent infiltration of CD8(+) T cells. In this regard, temporal and spatial distributions of PD-1(+) CD4(+) T cells might be susceptible to concurrently administered anti-PD-1.

On the other hand, the benefit of anti-PD-L1 was predicted by the expression of tumor-associated PD-L1 (B7-H1) by osteosarcoma cell lines. When confronted by tumor targets, EATs produce pro-inflammatory cytokines such as IFN-γ, upregulating PD-L1, which induces T cell apoptosis and inhibits T cell cytotoxicity [60]. Activation-induced T cell death (AICD), associated with IL-10 and Fas/FasL interaction [61], is a component of PD-L1-mediated T cell apoptosis and can be prevented by anti-PD-L1, but not by anti-PD-1 [60].

In addition, surface proteomic analysis of osteosarcoma has identified a wide range of proteins with differential abundance on osteosarcoma cells and human primary osteoblasts including ephrin type-A receptor (EPHA2) [62]. Using bioinformatics to assess the expression of surface target antigens on osteosarcoma could provide alternative promising strategy to discover new target antigens for T cell immunotherapies including BsAb and CAR [63, 64].

In conclusion, targeted T cell therapy using GD2-BsAb or HER2-BsAb enabled effective T cell infiltration into tumors and exerted potent anti-tumor activity against osteosarcoma*.* GD2-EATs and HER2-EATs were also effective to treat osteosarcoma xenografts with reduced toxicity. When GD2-BsAb and HER2-BsAb were combined with anti-PD-L1, tumors had more T cells inside, especially when anti-PD-L1 was continued post-GD2-BsAb treatment. These data strongly support the clinical applicability of GD2- and HER2-BsAbs and the sequentially continuous combination of anti-PD-L1 antibody for the treatment of osteosarcoma.

## Supplementary information

**Additional file 1**:** Table S1**. Purity, binding affinity and endotoxin of bispecific antibodies.

**Additional file 2**:** Figure S1**. (A) Representative flow cytometry analysis of tumor-associated target antigens in the osteosarcoma U-2 OS cell line. GD2, disialoganglioside; GD3, disialohematoside; HER2, human epidermal growth factor receptor 2; CSPG4, Chondroitin-sulfate proteoglycan 4; GPA, glycoprotein A33; L1CAM, L1 cell adhesion molecule; GPC-3, glypican-3; PSA, polysialic acid; PD-L1, programmed death-ligand 1; PSMA, prostate-specific membrane antigen; IGF2R; Insulin-like growth factor 2 receptor. Figure S2. (A) The geometric mean fluorescence intensities (MFIs) of GD2-BsAb and HER2-BsAb bound to EATs were measured using anti-idiotype or anti-human IgG Fc antibody. (B) Antibody-dependent T cell-mediated cytotoxicity assay (ADTC) using GD2-EATs and HER2-EATs at decreasing ET (effector to target) ratios and decreasing BsAb arming concentrations. (C) MFIs of GD2-EAT and HER2-EAT over time in flow cytometry. 1x10^6^ of T cells were armed with 0.5μg of GD2-BsAb (GD2-EAT) or HER2-BsAb (HER2-EATs) and measured the MFIs by APC-conjugated anti-human IgG Fc antibody. GD2-EATs and HER2-EATs were incubated at 4℃, and the MFIs of the live cells were analyzed at each time point. Figure S3. In vivo cytokine release by GD2-EATs. (A) Plasma TH1 cell cytokines including IL-2, IL-6, IL-10, TNF-α, and IFN-γ were measured after 4 hours of EAT treatment and compared among groups. (B) Plasma TH1 cell cytokine levels were analyzed at 4hrs, 12hrs, and 24 hours post-GD2-EAT treatment. The P values of AUC for plasma cytokine levels were analyzed. Figure S4. (A) Flow cytometry analyses of tumor infiltrating lymphocytes (TILs). (B) Comparison of TIL frequencies among groups treated with different combination schedules of anti-PD-1 antibody and GD2-EATs. (C) Comparison of the TIL frequencies among groups treated with different combination schedules of anti-PD-L1 antibody and GD2-EATs.

## Data Availability

All data generated or analyzed during this study are included in this published article or uploaded as supplementary information.

## Abbreviations

ATCs: Activated T cells
AICD: Activation-induced T cell death
ADTC: Antibody-dependent T cell-mediated cytotoxicity
AUC: Area under curve
BRG: BALB-*Rag2*^−^^/^^−^IL-2R-*γc*-KO
CAR: Chimeric antigen receptor
CSPG4: Chondroitin-sulfate proteoglycan-4
CT: Concurrent therapy
CRS: Cytokine release syndrome
GD2: Disialogangliosides
ET ratio: Effector-to-target cells ratio
EATs: Ex vivo BsAb-armed T cells
MFI: Geometric mean fluorescence intensity
GPC-3: Glypican-3
HMW-M: High molecular weight melanoma antigen
HER2: Human epidermal growth factor receptor 2
ICIs: Immune checkpoint inhibitors
IHC: Immunohistochemistry
IGF2R: Insulin-like growth factor 2 receptor
IL-11Rα: Interleukin 11 receptor-α
L1CAM: L1 cell adhesion molecule
PDXs: Patient-derived xenografts
PBMC: Peripheral blood mononuclear cells
PD-L1: Programmed cell death-1 receptor ligand-1
PD-1: Programmed cell death-1 receptor
PSA: Prostate-specific antigen
PSMA: Prostate-specific membrane antigen
ST: Sequential therapy
SCT: Sequentially continuous therapy
T-BsAbs: T cell engaging bispecific antibodies
TFH: T follicular helper cell
TILs: Tumor-infiltrating lymphocytes
