# Supplementary material for: GD2 or HER2 targeting T cell engaging bispecific antibodies to treat osteosarcoma
Source: J Hematol Oncol. 2020 Dec 10;13:172. doi: 10.1186/s13045-020-01012-y (PMC7731630; doi:10.1186/s13045-020-01012-y)
Supplement: Supplementary file 1 — Additional file 1: Table S1. Purity, binding affinity and endotoxin of bispecific antibodies. [file 13045_2020_1012_MOESM1_ESM.docx]

Supplementary Table S1. Purity, binding affinity and endotoxin of bispecific antibodies.

| Antigen | Antibody | Antibody format | Purity (%) | | Binding affinity, K_d_(M) | | Endotoxin (EU/mg) |
| --- | --- | --- | --- | --- | --- | --- | --- |
|  |  |  | HPLC | SDS PAGE | CD3 | Target antigen |  |
| GD2 | hu3F8 | IgG-[L]-scFv | 96.23 | 95.23 | 2.86E-09 | 3.61E-09 | <2 |
| HER2 | herceptin | IgG-[L]-scFv | 93.36 | 94.37 | 2.30E-09 | 1.34E-12 | <2 |
| CD33 | huM195 | IgG-[L]-scFv | 82.54 | 80.59 | 2.01E-08 | 2.467E-09 | <2 |
| GPA33 | huA33 | IgG-[L]-scFv | 91.06 | 84.94 | 3.56E-09 | 3.05E-10 | <2 |
| B7H3 | hu8H9-3.1 | IgG-[L]-scFv | 87.65 | 89.98 | 5.47E-09 | 9.75E-09 | <2 |
| CSPG4 | hu763 | IgG-[L]-scFv | 93.226 | 76.15 | 5.12E-09 | 4.35E-10 | <2 |
| L1CAM | hu3E7 | IgG-[L]-scFv | 95.99 | 95.72 | 6.15E-09 | 8.77E-10 | <2 |
| Lewis Y | hu3S193 | IgG-[L]-scFv | 92.969 | 86.4 | 4.17E-09 | 3.54E-08 | <2 |
