# Supplementary figures and images for: GD2 or HER2 targeting T cell engaging bispecific antibodies to treat osteosarcoma
Source: J Hematol Oncol. 2020 Dec 10;13:172. doi: 10.1186/s13045-020-01012-y (PMC7731630; doi:10.1186/s13045-020-01012-y)

Fig. S1.

(A)

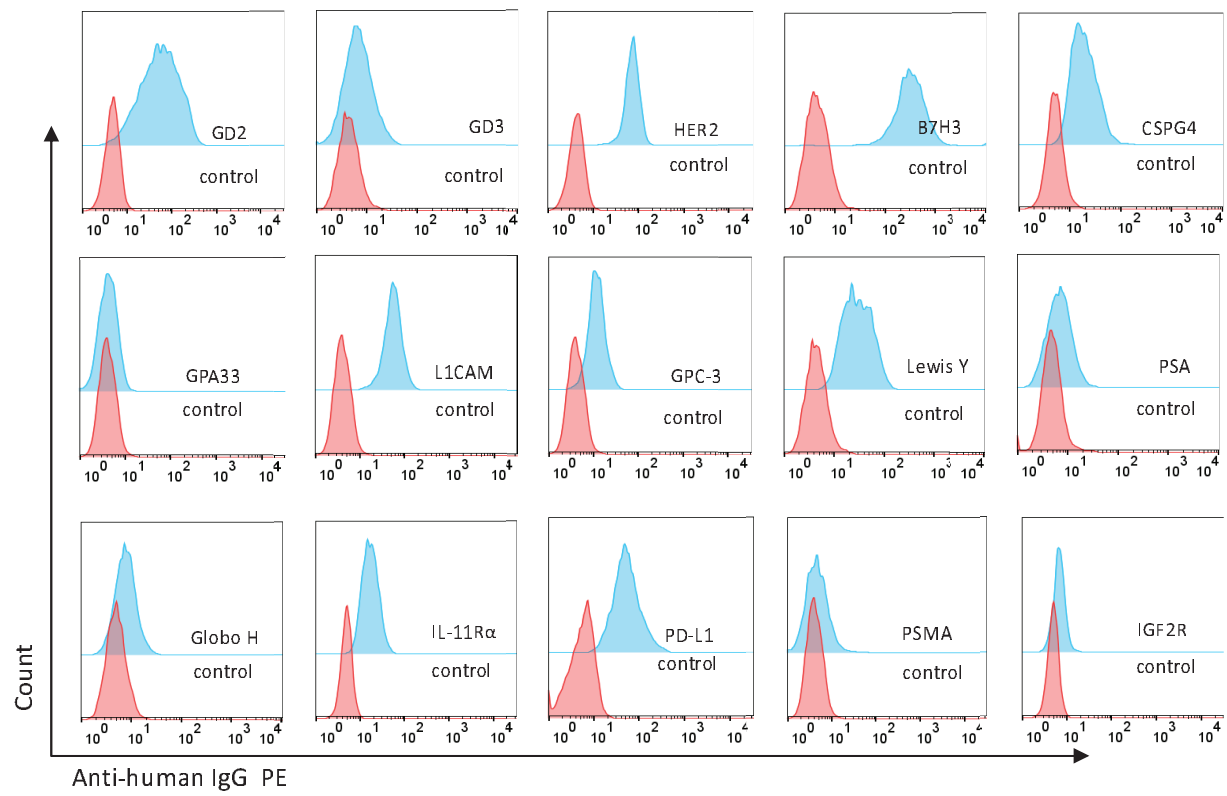

Fig. S2.

(A)

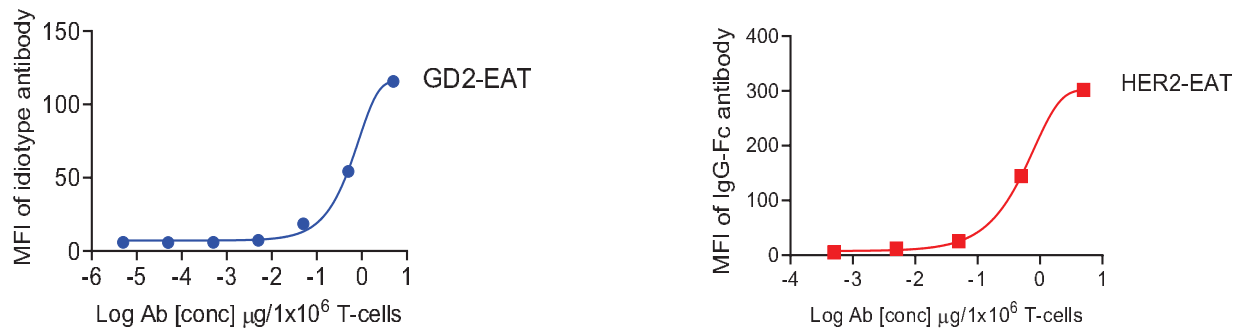

(B)

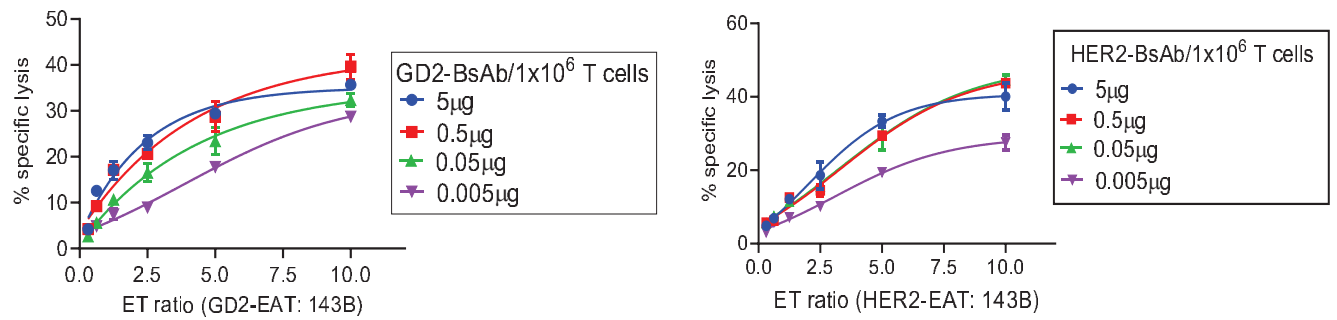

(C)

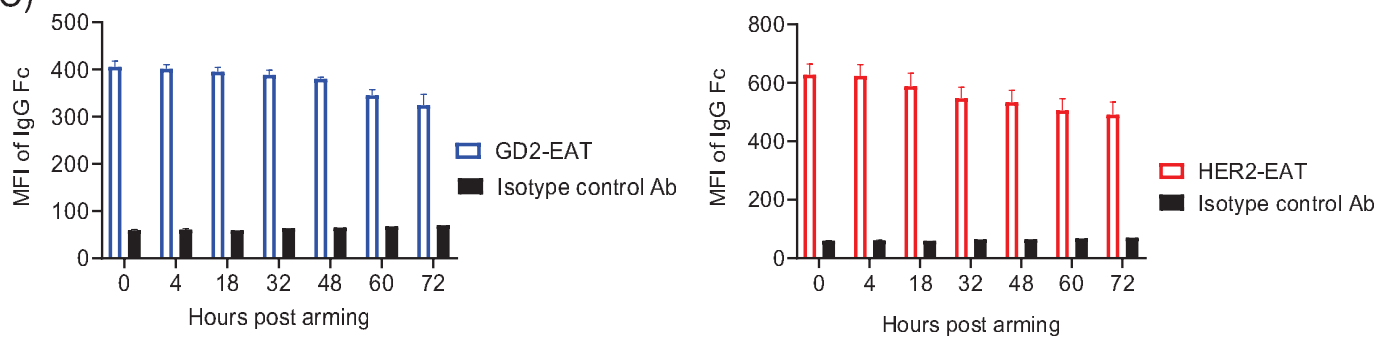

Fig. S3.

(A)

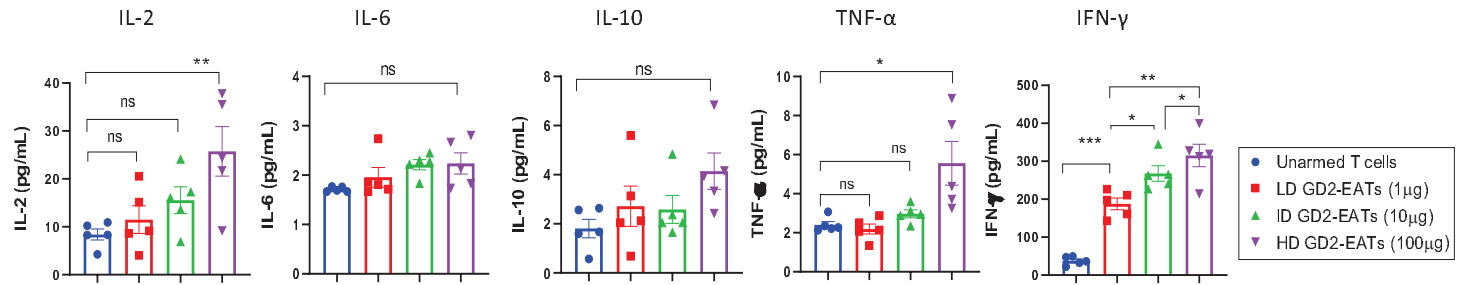

(B)

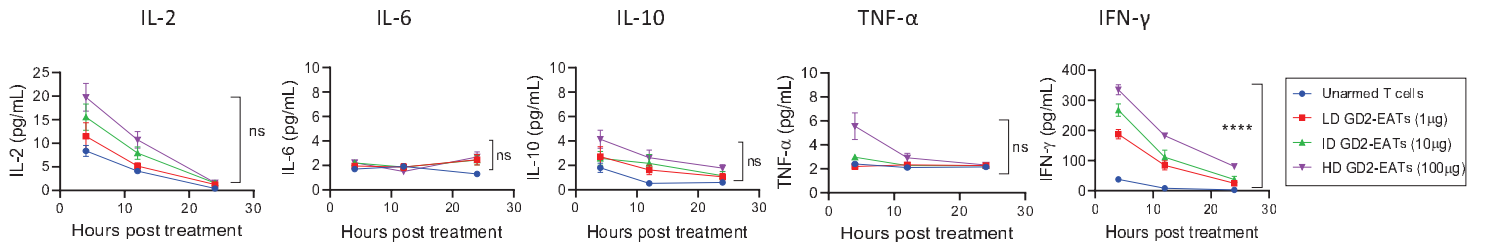

Fig. S4.

(A)

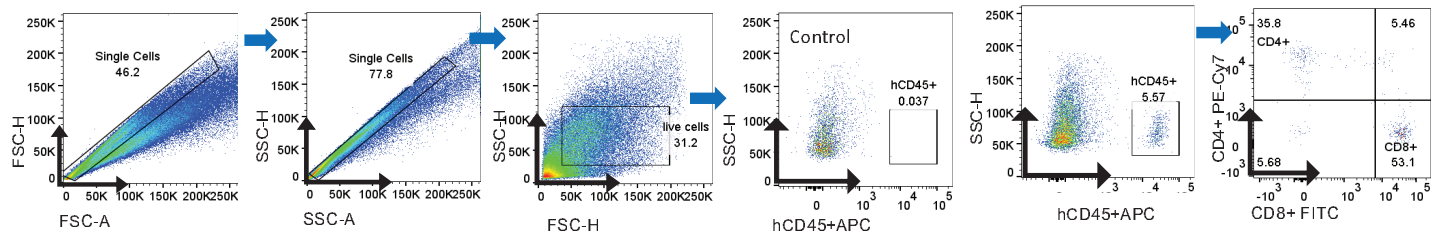

(B)

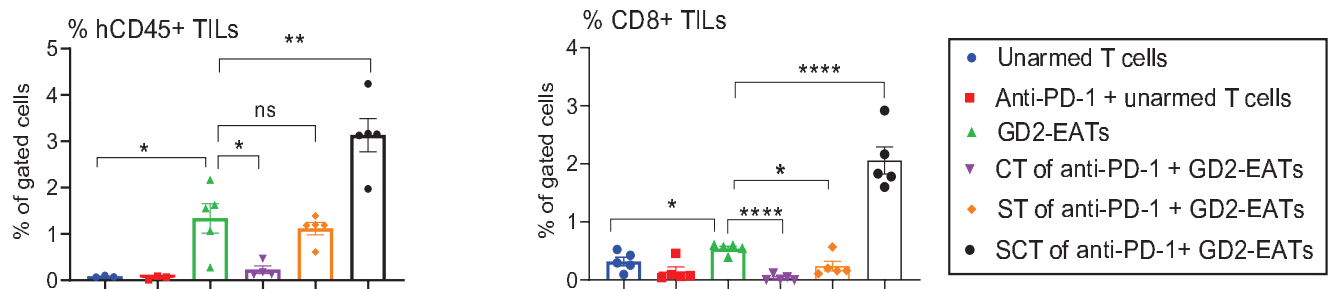

(C)

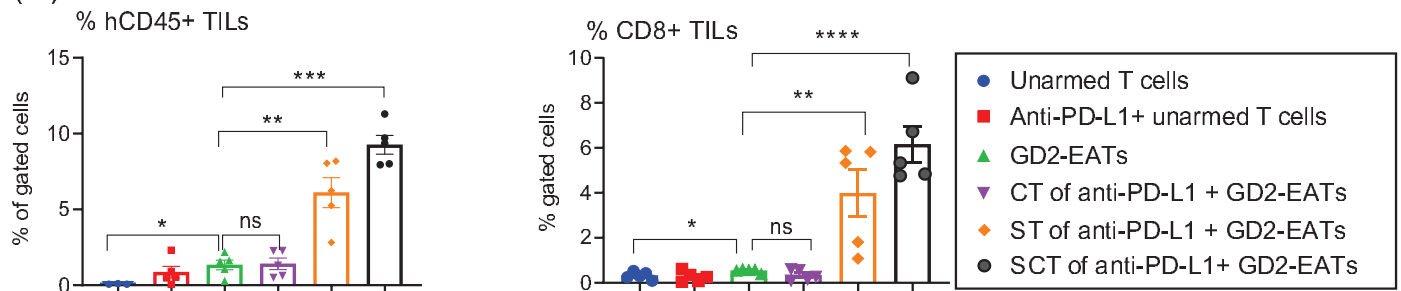

Supplement: Supplementary file 2 — Additional file 2: Figure S1. (A) Representative flow cytometry analysis of tumor-associated target antigens in the osteosarcoma U-2 OS cell line. GD2, disialoganglioside; GD3, disialohematoside; HER2, human epidermal growth factor receptor 2; CSPG4, Chondroitin-sulfate proteoglycan 4; GPA, glycoprotein A33; L1CAM, L1 cell adhesion molecule; GPC-3, glypican-3; PSA, polysialic acid; PD-L1, programmed death-ligand 1; PSMA, prostate-specific membrane antigen; IGF2R; Insulin-like growth factor 2 receptor. Figure S2. (A) The geometric mean fluorescence intensities (MFIs) of GD2-BsAb and HER2-BsAb bound to EATs were measured using anti-idiotype or anti-human IgG Fc antibody. (B) Antibody-dependent T cell-mediated cytotoxicity assay (ADTC) using GD2-EATs and HER2-EATs at decreasing ET (effector to target) ratios and decreasing BsAb arming concentrations. (C) MFIs of GD2-EAT and HER2-EAT over time in flow cytometry. 1x10^6^ of T cells were armed with 0.5μg of GD2-BsAb (GD2-EAT) or HER2-BsAb (HER2-EATs) and measured the MFIs by APC-conjugated anti-human IgG Fc antibody. GD2-EATs and HER2-EATs were incubated at 4℃, and the MFIs of the live cells were analyzed at each time point. Figure S3. In vivo cytokine release by GD2-EATs. (A) Plasma TH1 cell cytokines including IL-2, IL-6, IL-10, TNF-α, and IFN-γ were measured after 4 hours of EAT treatment and compared among groups. (B) Plasma TH1 cell cytokine levels were analyzed at 4hrs, 12hrs, and 24 hours post-GD2-EAT treatment. The P values of AUC for plasma cytokine levels were analyzed. Figure S4. (A) Flow cytometry analyses of tumor infiltrating lymphocytes (TILs). (B) Comparison of TIL frequencies among groups treated with different combination schedules of anti-PD-1 antibody and GD2-EATs. (C) Comparison of the TIL frequencies among groups treated with different combination schedules of anti-PD-L1 antibody and GD2-EATs. [file 13045_2020_1012_MOESM2_ESM.pdf]
